# Children’s and adolescent’s self - assessment of metabolic control versus professional judgment: a cross-sectional retrospective and prospective cohort study

**DOI:** 10.1186/1687-9856-2013-21

**Published:** 2013-12-17

**Authors:** Andreas Bieri, Monika Oser-Meier, Marco Janner, Chantal Cripe-Mamie, Kathrin Pipczynski-Suter, Primus E Mullis, Christa E Flück

**Affiliations:** 1 Pediatric Endocrinology, Diabetology & Metabolism, University Children’s Hospital, Inselspital, Freiburgstrasse 15, CH - 3010 Bern, Switzerland

**Keywords:** T1DM, Glycemic control, Self-assessment, HbA1c, Perception

## Abstract

**Background:**

Morbidity and mortality in T1DM depend on metabolic control, which is assessed by HbA1c measurements every 3–4 months. Patients’ self-perception of glycemic control depends on daily blood glucose monitoring. Little is known about the congruence of patients’ and professionals’ perception of metabolic control in T1DM.

**Objective:**

To assess the actual patients’ self-perception and objective assessment (HbA1c) of metabolic control in T1DM children and adolescents and to investigate the possible factors involved in any difference.

**Methods:**

Patients with T1DM aged 8 – 18 years were recruited in a cross-sectional, retrospective and prospective cohort study. Data collection consisted of clinical details, measured HbA1c, self-monitored blood glucose values and questionnaires assessing self and professionals’ judgment of metabolic control.

**Results:**

91 patients participated. Mean HbA1c was 8.03%. HbA1c was higher in patients with a diabetes duration > 2 years (p = 0.025) and in patients of lower socioeconomic level (p = 0.032). No significant correlation was found for self-perception of metabolic control in well and poorly controlled patients. We found a trend towards false-positive memory of the last HbA1c in patients with a HbA1c > 8.5% (p = 0.069) but no difference in patients’ knowledge on target HbA1c between well and poorly controlled patients.

**Conclusions:**

T1DM patients are aware of a target HbA1c representing good metabolic control. Ill controlled patients appear to have a poorer recollection of their HbA1c. Self-perception of actual metabolic control is similar in well and poorly controlled T1DM children and adolescents. Therefore, professionals should pay special attention that ill controlled T1DM patients perceive their HbA1c correctly.

## Background

The primary goal of diabetes care for children and adolescents is to achieve an optimal metabolic control to prevent or to minimize the risk of acute (e.g. hypoglycemia) and long-term complications such as retinopathy, nephropathy and neuropathy [1,2]. The recommended everyday treatment regimen for a patient with Type 1 Diabetes Mellitus (T1DM) is complex and demanding. Parents or other adult care takers initially play a key role in this intensive care system. But as children grow older responsibility for taking care of their chronic condition is placed upon them. During adolescence deteriorations in diabetes management and control are common [3]. These deteriorations raise the risk of acute or long-term complications and are also associated with higher health care costs. It is known that an optimal self-care behavior, independently of age, impacts positively on glycemic control [4]. Therefore, professionals aim to help adolescent patients and their families to become experts in self-management of their disease. A recently published systematic review investigated demographic and inter- or intrapersonal factors associated with metabolic control and self-care in adolescent patients with T1DM [4]. This revealed that adolescence is associated with both, decreased self-care and deterioration in metabolic control. Factors like a lower socioeconomic status, lower parental responsibility for, and involvement in diabetes-focused daily tasks, higher peer orientation or also intrapersonal characteristics like low conscientiousness and low emotional stability were associated with lower self-care and higher HbA1c values.

Self-care of diabetes in daily routine involves insulin administration, decisions around food – choices and intake, physical activity, timing of glucose measurements and analysis as well as response to the results. This calls for well-organized treatment instructions and continuous coaching by a multidisciplinary team but also for patients cognitive and executive skills. In recent years cognitive and executive functioning in T1DM gained attention in the literature [5,6]. These studies essentially showed only mild differences between the neurocognitive performance of children and adolescents with T1DM when compared to controls. In a meta-analysis of the literature in 2008 only a mildly reduced intellectual quotient was found in children with diabetes [5]. The largest effects, but still within a very small range, were on visuospatial ability, motor speed and writing, and on sustained attention and reading. Most of these investigations focused on cognition.

Overall, there is a body of knowledge about cognitive and executive functioning in T1DM and also of factors associated with self-care, adherence to therapy and metabolic control. By contrast, there is very limited knowledge about the T1DM patients’ capacity of self-assessment which obviously is a prerequisite for good self-care. Characteristics of self-assessment for example are self-perception of HbA1c value, patient’s memory of the HbA1c value, knowledge on target HbA1c or patients’ suggestions on how to improve metabolic control. We found only limited literature concerning the role that recall plays in diabetic management. Only very recently a study investigated the prospective recall and glycemic control in children with T1DM [7]. No clear association between glycemic control and memory was found. Similarly, no literature is available for the difference between patients’ and professionals’ assessment of metabolic control. Our daily experience suggests that patients’ self-assessment of the actual glycemic control depends primarily on the perception of their own diabetes management at home, including daily blood glucose self-monitoring, insulin applications and diet, whereas professionals’ assessment depends mainly on measured HbA1c levels and blood glucose measurements from home devices.

Therefore, the aim of our study was to test the hypothesis if there was a difference between patients’ self-perception and an objective assessment (HbA1c) of metabolic control in T1DM children and adolescents; and to investigate factors that may be involved.

## Methods

### Patients and study design

We performed a cross-sectional, retrospective and prospective cohort study. We recruited patients with T1DM, seen at the outpatient clinic of the University Children’s Hospital in Bern between April and September 2011. Inclusion criteria were an age between 8 – 18 years, diagnosis of T1DM for ≥ 12 months, at least 3 regular consultations in our department during the past 12 months and informed consent. Exclusion criteria were a change in the modality of insulin therapy in the past 12 months, less than 3 regular consultations in our department over the past 12 months, other chronic illnesses influencing the metabolic control of T1DM (such as malignancy or neuromuscular disease) and other types of diabetes. The study fulfilled the criteria of the Declaration of Helsinki and was approved by the cantonal ethics committee of Bern, Switzerland. Participating patients and caregivers were informed about the study and gave their written consent.

A total of 91 children (53 boys and 38 girls) were included in the study. 39 T1DM patients between 8 – 18 years did not participate for the following reasons: 3 refused to participate, 33 did not fulfill the inclusion criteria and 3 did not provide full information on the questionnaires. Details on patient characteristics are summarized in Table 1.

**Table 1:** Patient characteristics

**Number of patients (n)**	All	91	
	Male	53	
	Female	38	
		**Mean**	**Range**
**HbA1c (%)**	All	8.03	6.1 - 10.9
	Male	7.99	6.3 - 10.5
	Female	8.09	6.1 - 10.9
**Age (years)**		13.22	8.23 - 17.81
**Duration of T1DM (years)**	All	6.13	1.05 - 15.77
**Body mass index (SDS)**	All	0.06	-2.61 - 1.98
	Male	-0.10	-2.61 - 1.93
	Female	0.28	-1.65 - 1.98
		**n**	**%**
**Modality of therapy**	Conventional insulin therapy	9	9.9
	Functional insulin therapy	59	64.8
	Insulin pump	23	25.3
**Parental socioeconomic level**	Low	8	8.8
	Moderate	64	70.3
	High	17	18.7
	Not determined	2	2.2

### Data collection

Clinical and demographic data such as age, duration of disease, modality of insulin therapy and HbA1c of the last consultation were collected from patients’ clinical records. Height and weight were measured during the visit at the outpatient clinic. Standard deviation score of the Body Mass Index (BMI) was calculated according to the LMS model taking the Kromeyer-Hauschild percentiles as a reference [8].

Data concerning self-monitored blood glucose levels were taken from memory functions of personal glucometers. Average values per day were calculated over the past 2–4 weeks.

All other information was collected with the help of three specific questionnaires: One for the professional, one for the care taker and one for the patient. Patients were requested to fill in the questionnaires without the help of their care takers.

A classification of the socioeconomic level was performed based on the self-declared educational level and occupational status of both parents as published elsewhere [9]. In brief, the classification “low” consisted of public school without professional training; the “intermediate” level included secondary school with completed professional training, and a “high” level was defined as having completed academic studies at a university.

HbA1c was determined by the Latex-Immunagglutination method (DCA 2000 Analyzer, Bayer Corporation, Elkart, IN 46514 USA). For this assay, reference values for healthy, non-diabetic individuals range between 4.0 - 5.6%.

### Self assessment score (SAS)

We created a questionnaire and a scoring system to evaluate the quality of the self-assessment of patients’ metabolic control. Patients were asked by questionnaire whether they felt that the actual HbA1c might be better, equal or worse than the HbA1c measured 3 months ago. Better or worse were defined as a difference in HbA1c ≥ ^+^/_-_ 0.5%. Data were analyzed and categorized as follows. SAS 0 meant, that patient’s perception overlapped with the objective result. SAS +1 or +2 meant, that the measured HbA1c value showed an improvement which the patient did not perceive (e.g. the patient meant that the actual HbA1c was worse than the last HbA1c, but in fact it was equal (+1) or better (+2). SAS -1 or -2 meant, that the measured HbA1c value showed a worsening of the metabolic control which the patient did not realize (e.g. the patient meant that the actual HbA1c was equal or better than the last HbA1c, but in fact it was worse (-1 to -2).

### Data analysis

Data were analyzed using SPSS 19.0 (IBM® SPSS® Statistics 19). For group comparison the Kruskal-Wallis test was used. A p-value < 0.05 was considered to indicate statistical significance. Most data are shown as boxplots with the top of the box representing the 75^th^ percentile, the bottom of the box representing the 25^th^ percentile, and the line in the middle representing the 50^th^ percentile. The whiskers represent the highest and lowest values, that were not outliers or extreme values.

## Results

### Patient characteristics and metabolic control in the study cohort

Mean HbA1c of the 91 studied T1DM patients was 8.03% (range: 6.1 – 10.9%) (Table 1). In boys the mean HbA1c was 7.99%, in girls 8.09%. Mean duration of T1DM (time since the initial diagnosis) was about 6 years. Two thirds of the patients were treated with a functional insulin therapy using multiple daily injections, 25% of the patients with an insulin pump, and 10% were on a conventional 2–3 insulin injection regimen with fixed meals.

Two thirds of the patients had care givers classified as having a moderate level of socioeconomic status, 17% had a high and 8% a low level.

**Figure 1:**
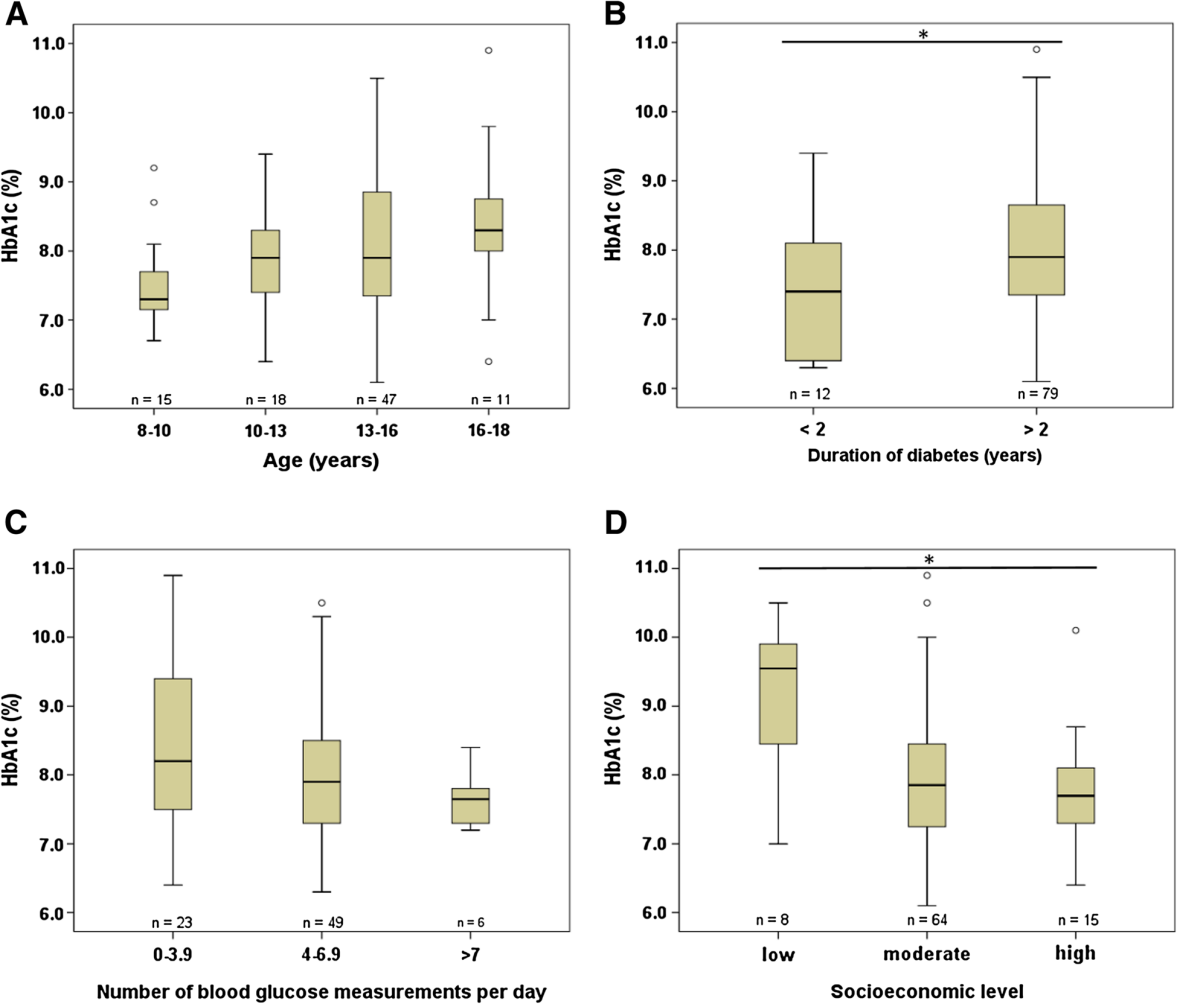
**HbA1c in relation to (A) age, (B) duration of diabetes, (C) glucose self-monitoring and (D) socioeconomic level.** There is a tendency towards higher HbA1c values with age (p = 0.065). HbA1c values correlate with the duration of diabetes (p = 0.025). HbA1c does not correlate with the number of blood glucose self-measurements (p = 0.173) but correlates with the socioeconomic level (p = 0.032). Data are given as boxplots and were statistically analyzed by Kruskal-Wallis tests with a significance level of p ≤ 0.05.

Figure 1 shows the HbA1c values in the study cohort in relation to age, duration of diabetes, glucose self-monitoring and socioeconomic level. We found significant correlations between HbA1c values and the duration of diabetes, with higher HbA1c values in patients with diabetes for > 2 years. Similarly, HbA1c values were significantly higher in the lowest socioeconomic group as compared to the moderate and high socioeconomic group (Figure 1D). Finally, we observed a trend towards higher HbA1c values in older patients (p = 0.065).

### Memory of the HbA1c measured at the last consultation

**Figure 2:**
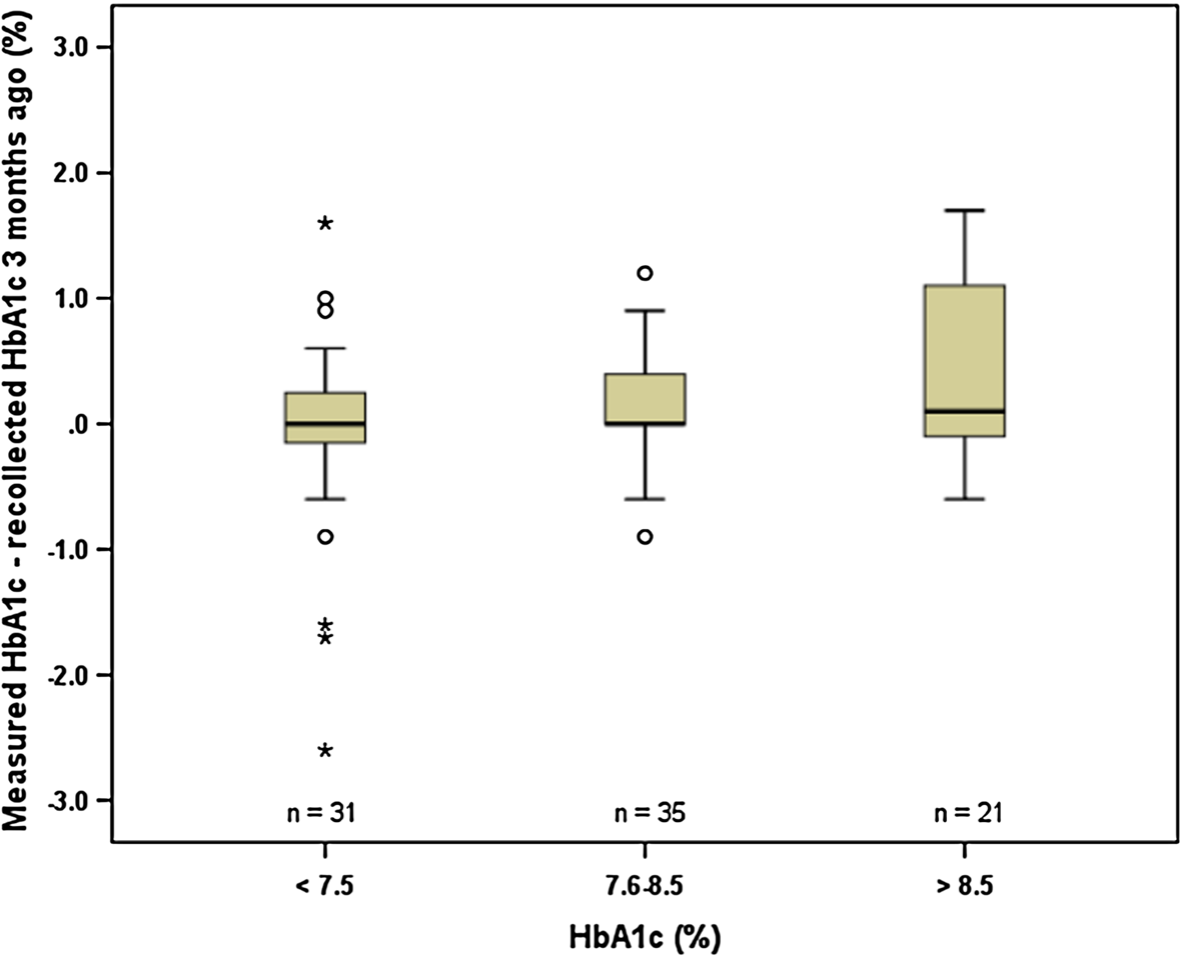
**Memory of last HbA1c.** The recollection of the last measured HbA1c values was assessed by comparing the objective HbA1c values 3 months ago with the patient’s recollection of this HbA1c. The gap between the last measured and remembered HbA1c value was then compared to the actual HbA1c. Data are shown as boxplots with the actual HbA1c in categorized form on the x-axis. Note that there is a tendency towards wrong positive memory of the last HbA1c in patients having an HbA1c > 8.5% (p = 0.069). Data were analyzed by the Kruskal-Wallis test with a significance level of p ≤ 0.05.

To investigate the impact of regular consultations with diabetes professionals at our center, the memorized HbA1c of the last visit was studied. Recollection of the HbA1c measured during the former visit 3–4 months ago was assessed by questionnaire and compared with the HbA1c value from the laboratory. The difference between the recalled and the measured HbA1c were then compared to the actual HbA1c, age, frequency of blood glucose self-monitoring, duration of diabetes and socioeconomic level. We found that patients with HbA1c values > 8.5% tended to have a poorer recollection of their last HbA1c than better controlled subjects (p = 0.069) (Figure 2). By contrast no relationship was found between the anamnestic HbA1c and age, frequency of glucose self-monitoring, duration of diabetes and socioeconomic level (data not shown).

### Knowledge of target HbA1c

**Figure 3:**
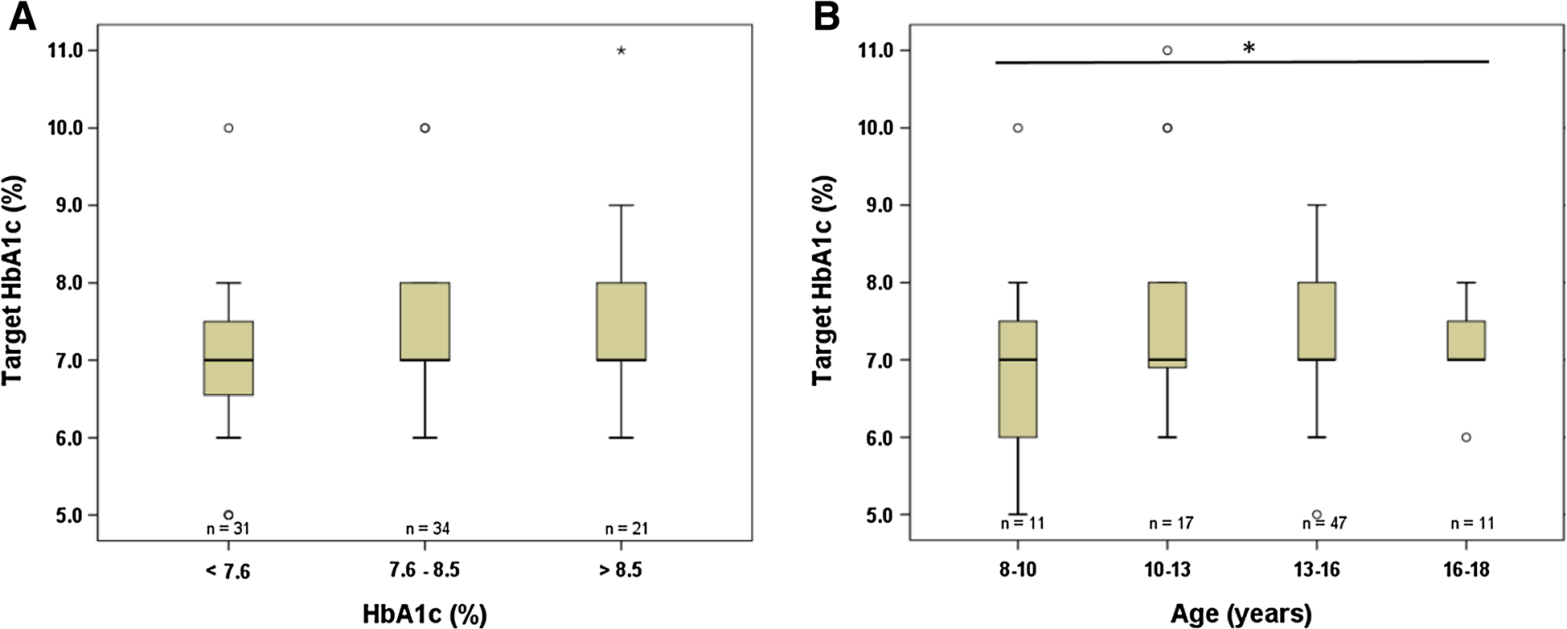
**Knowledge of target HbA1c.** All patients were asked for the currently recommended HbA1c level for good glycemic control (y-axis). **A)** These data were then compared to the actual HbA1c of each patient (x-axis). No significant difference was found (p = 0.154). **B)** Data were also correlated with the age finding significantly higher target HbA1c levels in older patients (p = 0.017). Data were analyzed by the Kruskal-Wallis test with a significance level of p ≤ 0.05.

Quality of metabolic control in diabetic patients is followed by regular HbA1c measurements. Internationally a target HbA1c of < 7.5% is recommended for all age groups [10]. This basic information on diabetes is conveyed to our patients and parents/caregivers by our team during initial instructions and is part of the communication during every follow-up visit. Therefore, we asked our patients for their target HbA1c and then correlated this value with their measured HbA1c value at the time of the visit, age, blood glucose self-monitoring, duration of diabetes and socioeconomic level (Figure 3). Overall, we found no relationship between knowledge of target HbA1c and measured HbA1c (Figure 3A). By contrast, older patients indicated higher target values of HbA1c than younger patients (p = 0.017) (Figure 3B). No relationship was found between target HbA1c and the frequency of glucose self-monitoring, duration of diabetes and socioeconomic level (data not shown).

### Self-perception of metabolic control in T1DM

To assess our patients’ self-perception of their metabolic control, we invited them to predict whether the current measured HbA1c would be better, same or worse than the HbA1c assessed during the prior visit. Data were scored (SAS) and related to the actual HbA1c, age, frequency of blood glucose self-monitoring, duration of diabetes and socioeconomic level. For details concerning the SAS see the Methods section. Generally, patients with a SAS of 0 had a perfect fit between their prediction and the actual HbA1c measurement, while patients with a SAS of +/-2 had the biggest difference between their prediction and the objective measurement.

**Figure 4:**
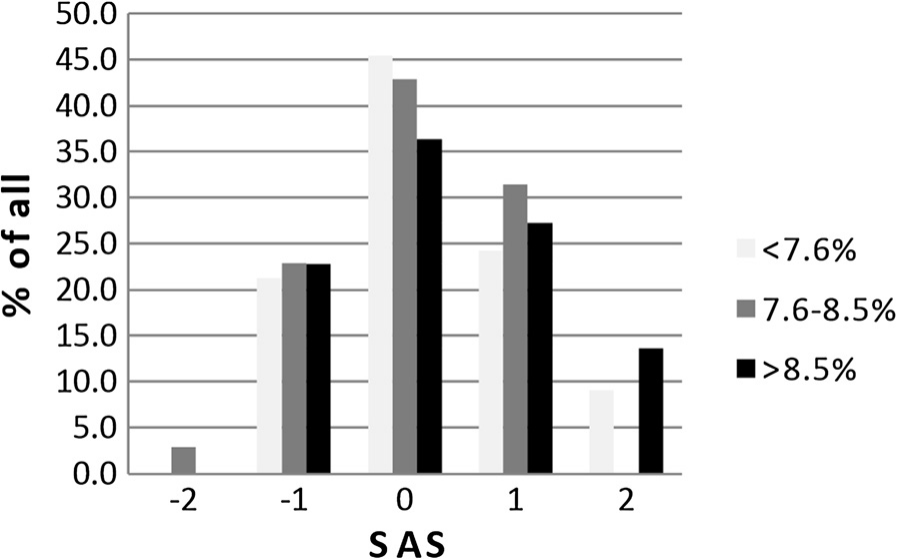
**Self-perception of metabolic control in T1DM.** HbA1c levels were put in relation to a self assessment score (SAS). Patients were asked to predict their HbA1c qualitatively. Data were collected with questionnaires and categorized from -2 to +2. A SAS 0 meant that patient’s perception overlapped with the objective result. A SAS of +1 or +2 meant that the measured HbA1c value was better than the last one but this improvement was not perceived by the patient. A SAS -1 or -2 meant that the actual HbA1c value was worse than the last one but predicted otherwise by the patient. No significant correlation was found between the SAS and the actual HbA1c level (p = 0.99). Data are shown as bar graphs and were analyzed by the Kruskal-Wallis test.

We found that nearly half of the patients with a HbA1c value < 7.6% had a perfect fit showing a SAS of 0, whereas only 36% of the patients with a HbA1c value > 8.5% had a SAS of 0 (Figure 4). However, this effect was not significant (p = 0.99). There was a trend, that patients with a longer duration of diabetes overestimated their actual HbA1c false-positively (p = 0.095) (data not shown).

Interestingly, the largest proportion of patients predicted their metabolic control correctly irrespective of their actual HbA1c (36-45%) while only few made a grossly wrong prediction (Figure 4).

### Suggestions for improving metabolic control

**Figure 5:**
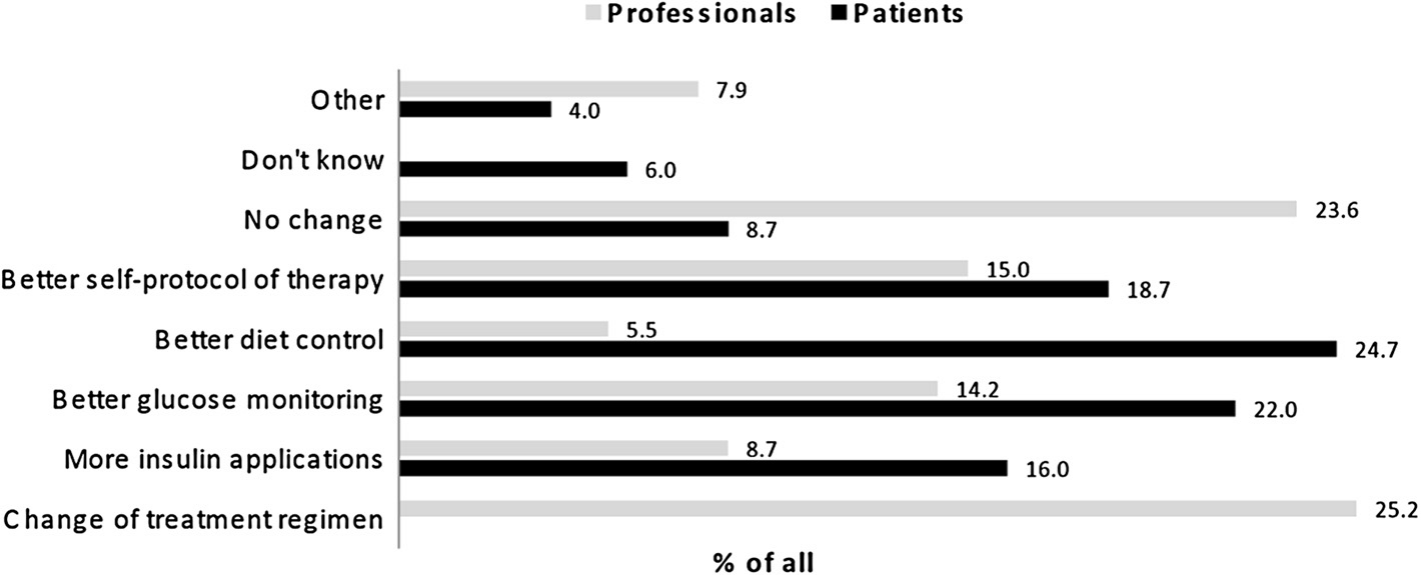
**Comparison between professionals’ and patients’ suggestions to improve metabolic control in T1DM.** Professionals and patients were invited to make suggestions to improve or maintain metabolic control. A list of items was given. Professionals and patients could choose one or more of the listed items. Only professionals had the possibility to choose the item *Change of treatment regimen* while only patients could choose the answer *Don’t know*. Data were analyzed descriptively and are shown as % of all.

Professionals and patients were invited to make suggestions on how to improve or maintain metabolic control. A list of items was given. Data were analyzed descriptively and results are shown as percentage (Figure 5). Professionals often suggested a *Change of the treatment regimen* or *No change*. By contrast, patients more often suggested a change in their daily routine like *Intensified glucose monitoring*, *Modification of nutrition* or *More elaborate self-protocol of therapy*.

Additional analysis revealed a relationship between the number of daily measurements of blood glucose and the age, with a higher number of daily measurements of blood glucose in younger patients (p = 0.012), who also tended to have lower HbA1c levels. On average patients in the age category of 8 – 10 years (n = 12) performed 5.3 glucose self-measurements daily, patients in the age category of 10 – 13 years (n = 16) 5.7, patients in the age category of 13 – 16 years (n = 41) 4.6 and patients in the age category of 16 – 18 years (n = 9) 3.4 only.

Our study questionnaire also included a query concerning the most annoying thing in the patients daily diabetes care: “If you could skip something in your daily diabetes care, what would it be?” We suggested the following items: *Insulin injections*, *Glucose measurements*, *Self-protocol of the therapy in a booklet or electronic device, Diet issues* or *Other.* From a total of 87 answers, 39% (n = 34) chose the answer *Insulin injections,* 37.9% (n = 33) chose *Self-protocol of the therapy in a booklet or electronic device,* 10.9% (n = 9) answered with *Glucose measurements,* 6.9% (n = 6) were annoyed with *Diet issues* and 5.7% (n = 5) chose *Other* issues including regular change of catheters of insulin pump or drawing venous blood for recommended laboratory control once a year. When we related these answers to the age of the patients, we observed, that older patients were especially annoyed at having to self-protocol the therapy in a booklet or electronic device and at glucose self-measurements, while younger patients would rather skip insulin injections or diet issues.

## Discussion

This study in a small cohort of a single center shows that self-perception of metabolic control is good in children and adolescents with T1DM irrespective if well or poorly controlled.

Little is known about T1DM patients’ capacity to self-assess therapy. This includes self-perception of HbA1c value, patient’s recall of the HbA1c value, knowledge of target HbA1c level as well as patients’ suggestions on how to improve metabolic control. This is in contrast to good knowledge on neurocognitive functioning in T1DM patients and of factors associated with self-care, adherence to therapy and metabolic control.

We found that the self-perception of actual metabolic control is similar in well or poorly controlled T1DM patients. This raises the question what factors influence metabolic control, and what factors influence the ability of self-assessment. It is well known, that for example the frequency of blood-glucose self-monitoring, the age of the patients, the duration of disease or the socioeconomic background influence metabolic control [2,11]. Therefore, we wondered whether these same factors were also associated with the ability of self-assessment of metabolic control.

In general, our patients with T1DM have a satisfactory metabolic control with a mean HbA1c of 8.03%. This compares to a cross-sectional study from our center in 2008 with a mean HbA1c of 7.6% [12]. The difference in HbA1c may be explained by the fact that in the study in 2008 all T1DM patients aged 0 – 20 years were enrolled without further limitations. In this study a large proportion (69%) of the patients had a short diabetes duration of 0 – 24 months with presumed residual activity. In line with the actual study the subgroup of diabetic adolescents also had a mean HbA1c of 8.1%. Compared to a large, international multicentre study which reported a mean HbA1c of 8.2% [13], our results are slightly better. Similar to other studies [2,14], we show that metabolic control is better with shorter duration of diabetes and higher socioeconomic level.

We found, that poorly controlled patients (HbA1c > 8.5%) have a worse recollection of their last HbA1c compared to better controlled subjects. Only one recent study investigated the prospective memory in correlation with glycemic control in children with T1DM [7]. Prospective memory was defined as the memory which is required to carry out intended actions. This study employed PROMS, an innovative prospective memory screen and a series of cognitive tests. Overall, this was a largely negative study which found no association between total PROMS score and glycemic control. Most studies investigating neurocognitive functioning in pediatric T1DM patients conclude that severely low blood glucose levels increase the risk of learning difficulties and a range of cognitive deficits and memory function [5,6]. As we found that poorly controlled patients have a worse recollection of their last HbA1c, we assumed that they overestimated their metabolic control in personal favor. In fact, false-positive recollection of metabolic control can harm the diabetic patient because no actions will be taken to achieve euglycemia (including insulin dose adjustments, intensified glucose monitoring, and diet control). Therefore, regularly measured HbA1c and discussions with professionals are strongly recommended to prevent wrong self-assessment. Factors like age, frequency of glucose self-monitoring, duration of diabetes and socioeconomic level alone don’t seem to correlate with the ability of memorizing the personal HbA1c level.

In regards to the knowledge about target HbA1c, no correlation was found with metabolic control. By contrast the personal target HbA1c level correlated with age, with higher personal target levels in older patients. This is inconsistent with the findings of the Hvidoere Childhood Diabetes Study 2005 [13], where reported target HbA1c levels were associated with the actual metabolic control, but not associated with age. The fact that target levels in our study did not correlate with metabolic control, is probably due to the small number of patients in our study. The observation that our older T1DM patients have a higher target HbA1c in mind remains unexplained. It has been reported that, if members of the diabetes care team are consistent in their advice on target HbA1c, adolescents’ HbA1c correlates with those targets [13]. So we presume that it is an important teaching point in diabetes care, that patients are aware of the internationally recommended target HbA1c, which is < 7.5% for all age groups [10].

Furthermore, it is discussed in the literature that lower HbA1c levels and longer duration of diabetes might be factors that increase the risk for hypoglycemia in children and adolescents with diabetes [13,15,16]. Therefore, it is conceivable that higher HbA1c levels or a higher personal target HbA1c level might result out of fear of hypoglycemic episodes, especially in patients with hypoglycemia unawareness or recurrent severe hypoglycemia. However, the question whether frequent and/or severe hypoglycemic episodes affect T1DM patients’ self-perception of metabolic control is not solved in the literature and remains unsolved as we did not record hypoglycemic episodes for analysis in our study.

We found no correlation between the self-assessment score (SAS) and the actual measured HbA1c or other parameters. Interestingly, the largest proportion of patients in our study predicted their metabolic control correctly irrespective of their actual HbA1c. This may result from the therapeutic approach of our diabetes team to discuss the actual metabolic control with patients and parents and try to support patients in their efforts to improve metabolic control with personal advice. Currently there is no literature to compare these findings. When we assessed professionals’ and patients’ suggestions to improve actual metabolic control, we found that professionals often suggested a change of treatment regimen or no change, while patients rather suggested changes in their daily routine at home, like improving glucose monitoring or self-protocol or adapting nutrition. This reflects the different perspectives on diabetes management between professionals and patients well. While professionals are primarily preoccupied with values of HbA1c, glucose and insulin doses, patients deal with blood glucose self-monitoring, insulin applications and their diabetes diet regimen and know about their personal compliance. The ideal professional diabetes care has to integrate these two perspectives to reach consensus on what needs to be done to achieve good metabolic control. This goal may only be achieved with a multidisciplinary specialist team consisting of psychologists, social workers, dieticians, diabetes nurse instructors and pediatric diabetologists. Partners of the team may also be pediatricians, teachers or day-care professionals.

Interestingly, when focusing on the answers of the patients concerning our question of the most annoying thing in their daily diabetes care, we found that our patients are just as annoyed by insulin injection as to having self-protocol the therapy in a booklet or electronic device. Especially the older patients were annoyed at the continuous task of keeping a diary. There is hope, that further development of electronic devices will facilitate and simplify patients’ self-protocol of therapy in the future.

## Conclusion

Self-perception of metabolic control in children and adolescents with T1DM treated according to international standards is good, even if the objective metabolic control does not meet the target. Patients with poor metabolic control are less attentive to their actual HbA1c. In theory, T1DM patients know the target HbA1c levels for excellent metabolic control. Overall, current diabetes care strategies seem to achieve the goal to make T1DM patients experts of their own diabetes.

## Abbreviations

T1DM: Type 1 Diabetes Mellitus; IDF: International Diabetes Federation; ISPAD: International Society for Pediatric and Adolescent Diabetes; BMI: Body mass index; LMS: Least mean square; SAS: Self assessment score.

## Competing interests

The authors declare that they have no competing interests.

## Authors’ contribution

The authors’ contribution to the paper is as follow: AB and CEF: study concepts and design, data analysis and interpretation, statistical analysis, critical revision of the manuscript for important intellectual content and manuscript preparation; MOM, CCM, KPS, PEM: acquisition of data, critical revision of the manuscript for important intellectual content: MJ: study concepts and statistical analysis. All authors read and approved the final manuscript.
